# The Army Surgeon’s Manual

**Published:** 1865-09

**Authors:** 


The Army Surgeon's Manual, for the use of Medical Officers, Cadets, Chaplins, and Hospital Stewards, containing the Regulations of the Medical Department, all General Orders from the War Department, and Circulars from the Surgeon-General's Office. From January 1, 1861, to April 1, 1865. By William Grace, of Washington, D.C. Second edition. Published by permission of the Surgeon-General. New York: Balliere Bros., 520 Broadway. 1865.

This is a small sized octavo volume, of 225 pages. It is divided into four parts. The first part contains a list of the Medical Staff of the United State's Army, in February, 1865.

The second part contains the "Regulations of the Medical Department, from the Revised Regulations for the Army."

The third part contains the "General Orders relative to the Medical Department."

The fourth part contains Circulars of instruction, &c. The work having been published by permission of the Surgeon-General, and reached a second edition, we presume its statements and instructions are correct. It is designed more especially for medical officers of the Army, but will be found useful, for reference, by all classes.

